# Antiepileptic Therapy of *Abrus cantoniensis*: Evidence from Network Pharmacology

**DOI:** 10.1155/2022/7748787

**Published:** 2022-06-06

**Authors:** Yue Wang, Xia Li, Peixuan Dou, Tong Qiao, Ying Chang

**Affiliations:** ^1^Changchun University of Chinese Medicine, Postgraduate of College of Chinese Medicine, 130117, No. 1035, Boshuo Road, Jingyue National High-tech Industrial Development Zone, Changchun, Jilin (Province), China; ^2^Affiliated Hospital of Changchun University of Chinese Medicine, Chief Physician of Neurology, Master Tutor, 130000, Gongnong Road, Changchun, Jilin, China; ^3^Middle School, Changchun, Jilin (Province), China

## Abstract

The present study explores the mechanism of antiepileptic treatment of *Abrus cantoniensis* through network pharmacology. During this process, several databases were recruited, e.g., the TCMSP database, HERB database, and SwissTargetPrediction database were used to retrieve the active components and targets of *Abrus cantoniensis*; GeneCards database and OMIM database were used to retrieve the targets of epilepsy. The targets of epilepsy and *Abrus cantoniensis* were subjected to target intersection in venny2.1, and protein interaction analysis of *Abrus cantoniensis* in the String database. We set the Cyto NCA plug-in condition as betweenness; selected the first 8 genes of betweenness as the core genes; performed the integrative bioinformatics of candidates by GO analysis and KEGG analysis. Moreover, AutoDockTools and AutoDockVina software were used to perform the molecular docking; Pymol was used to perform the docking visualization. We obtained three active components of *Abrus cantoniensis*, which are mainly related to *β*-sitosterol and stigmasterol; 92 intersection targets of epilepsy of *Abrus cantoniensis*, including 9 core targets such as AKT1, ESR1, MMP9, CES1, SRC, HIF1A, ABCB1, CASP3, and SNCA; 8 core targets were flavanone constituent proteins. Define *p* value less than 0.05; according to the screening principle, the first 20 GO pathways and KEGG pathways were selected. We found that *Abrus cantoniensis* was mainly connected with epilepsy through the neuroactive ligand-receptor interaction signaling pathway, the neurodegeneration pathway, and multiple disease signaling pathway; the docking between ESR1 and components is the most stable among the core targets. Besides, the binding energies of the core targets were all less than −5 kcal mol^−1^. Taken together, the current research provides a new strategy for the antiepileptic treatment of Abrus cantoniensis.

## 1. Introduction

Epilepsy is a chronic noninfectious disease caused by paroxysmal abnormal electrical activity of brain neurons, which may affect people of all ages [1]. It is also known as one of the most common neurological diseases globally, characterized by repeated seizures and unprovoked seizures [2]. The clinical symptoms of epileptic seizures are complex and diverse, distinguished by paroxysmal motor, sensory, autonomic nerve, consciousness loss, and mental disorders. To cure epilepsy, patients should comprehensively adopt various treatment methods and carefully weigh their advantages and disadvantages. The long course and difficult epilepsy treatment often lead to a decline in the quality of life, e.g., a heavy economic burden to individuals, families, and society [3]. Nowadays, epilepsy treatment mainly includes drug therapy, i.e., select antiepileptic drugs according to different seizure types; physical therapy, i.e., vagus nerve stimulation, stereotactic radiotherapy, deep brain electrical stimulation; gene therapy; ketogenic diet; and surgical treatment [4, 5]. Among them, antiepileptic drugs are the first choice. However, long-term Western medicine treatment incurs adverse reactions such as nausea, vomiting, liver and kidney function impairment, and even cognitive dysfunction [6]. In addition, not all patients can tolerate Western medicine, and noncompliance with medical advice is inevitable, so long-term use of Western antiepileptic medicine is impossible, limiting its effect on the treatment of epilepsy. Hence, we need an efficient, safe, and cheap drug to treat epilepsy.

**Figure 1:**
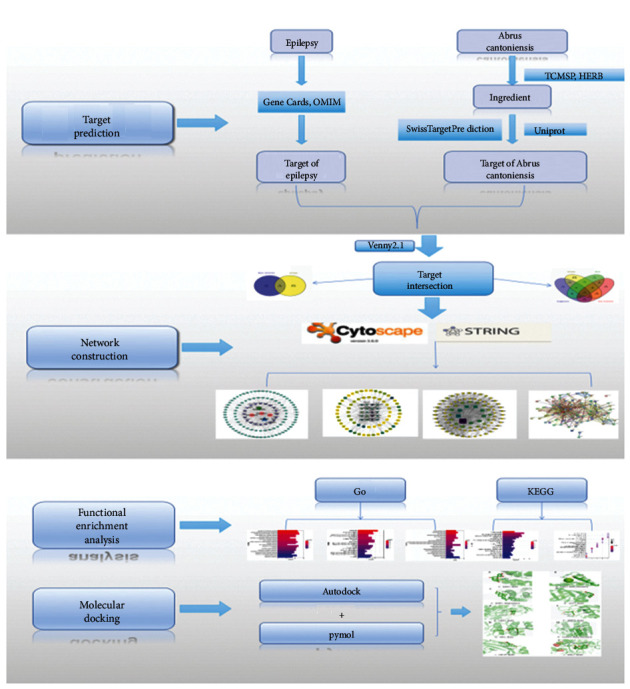
The flow chart of the article: the first part collects *Abrus cantonensis* and epilepsy genes. The second part is the mapping between *Abrus cantoniensis* gene and epilepsy gene, network diagram construction, pathway enrichment, and core target screening. The third part is the docking of core targets and effective components.

Traditional Chinese medicine (TCM) has a long-lasting efficacy and small side effects in treating epilepsy. The Chinese used them thousands of years ago to cure diseases. *Abrus cantoniensis*, a traditional Chinese medicine, is sweet, slightly bitter, and cool. After liver and stomach meridians enter, it merits clearing away heat and toxic materials, soothing the liver, and relieving pain. It also helps diuresis and jaundice and is often used to smooth symptoms such as jaundice, milk carbuncle, hypochondriac discomfort, and stomach ache. Despite all these advantages, one should be cautious when taking it: the seeds of this medicine are poisonous, so its pods should be removed to prevent poisoning. Studies have shown that *β*-sitosterol, the affective component of *Abrus cantonensis*, has no toxicity to primary rat hippocampal neurons and has a therapeutic effect on epilepsy [7]. However, the mechanism of this drug's effectiveness was not systematically described. To achieve this goal, we employed the methodology of network pharmacology to explore the effective active ingredients and potential targets of *Abrus cantoniensis* and discussed its possible antiepilepsy mechanism (see the research flow chart in Figure 1).

## 2. Materials and Methods

### 2.1. Predict Active Ingredients of *Abrus cantoniensis*

The effective ingredients were obtained using “*Abrus cantoniensis*” as the keyword, searching through TCM systematic pharmacology technology platform (TCMSP, https://tcmspw.com/tcmsp.php) and HERB database (https://herb.ac.cn/) under specific criteria, e.g., pharmacokinetics [8], oral bioavailability (OB)≥30%, and drug-likeness (DL)≥0.18. Also, the main effective active ingredients and targets of *Abrus cantoniensis* were screened during this process.

### 2.2. Acquire and Collect Action Targets of *Abrus cantonensis*

The TCMSP database and SwissTargetPrediction database were used to predict the target of related active ingredients first, and the target of active ingredients of *Pinellia ternata* was obtained afterward by importing it into the Uniprot online protein database (https://www.uniprot.org/).

### 2.3. Acquire Epilepsy-Related Targets and Map with *Abrus cantoniensis* Prediction Targets

The epilepsy-related targets were obtained using “epilepsy” as a keyword and searched through the GeneCards database (https://www.genecards.org/) and OMIM database (https://omim.org/). Following, the duplicate target genes were deleted. Finally, both genes of epilepsy and *Abrus cantoniensis* were mapped together to get a venny map.

### 2.4. Protein-Protein Interaction (PPI) Network Analysis and the Core Targets Screen

The potential target of *Abrus cantonensis* for epilepsy treatment was introduced into the String database (https://string-db.org/), and the species “*Homo sapiens*” was selected to obtain a PPI network. The data was then imported into the Cytoscape 3.9.0 software. The first 8 genes in-betweenness were selected as the core targets using the CytoNCA plug-in's betweenness.

### 2.5. GO Analysis and KEGG Pathway Enrichment Analysis

The overlapped targets were imported into the Metascape database (https://metascape.org/gp/index) for Gene Ontology (GO) analysis and Kyoto Genomics and Genomics Encyclopedia (KEGG) analysis; the parascreening meter was set as the false discovery rate (FDR) < 0.05. Then, import KEGG path relationship file into the Cytoscape 3.9.0 software to predict the degree value, which was used to adjust the node size afterward. Finally, the “target -KEGG path” relationship network diagram was generated.

### 2.6. Construct and Analyze “Drugs-Active Ingredients-Diseases-Targets” Network

The obtained potential targets, KEGG pathways, diseases, and *Abrus cantoniensis* for epilepsy treatment were sent to the Cytoscape software to construct a “drug-active ingredients-diseases-targets” regulatory network diagram. Then, analyse the potential relationship between genes and components of *Abrus cantoniensis* by a network diagram.

### 2.7. Dock Component-Target Molecule

Molecular docking of the screened core target and its corresponding active components was carried out to verify the binding activity between components and targets. Firstly, the protein crystal structure of the core target was downloaded from the RCSB PDB database (https://www1.rcsb.org/) and used PyMol to remove the original ligand and water molecule. In addition, the components were introduced into ChemBio3D Ultra for energy minimization. Then, AutoDockTools was used for file conversion before docking, and AutoDockVina was used followed for molecular docking. Finally, the lowest free energy sample was selected as the docking sample and visualized by PyMol.

## 3. Result

### 3.1. Screen Active Ingredients of *Abrus cantoniensis*

46 active ingredients of *Abrus cantoniensis* were searched based on the TCMSP database. We set OB ≥ 30% and DL ≥ 0.18 to obtain three main active ingredients of *Abrus cantoniensis*, including *β*-sitosterol, stigmasterol, and 3-hydroxyflavanone (see Table 1).

### 3.2. Predict Potential Targets of *Abrus cantoniensis* Components in Epilepsy Treatment

**Figure 2:**
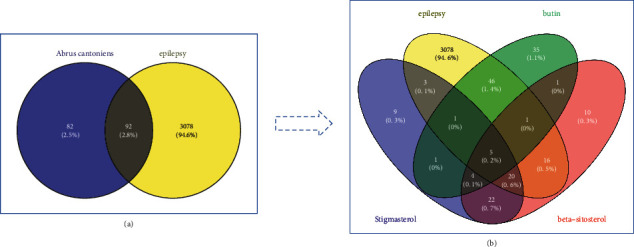
(a) Venny diagram, yellow represents targets of epilepsy; blue represents targets of *Abrus cantonensis*; gray represents the intersection gene between *Abrus cantonensis* and epilepsy. (b) Intersection targets of epilepsy with *β*-sitosterol, stigmasterol, and 3-hydroxyflavanone.

**Figure 3:**
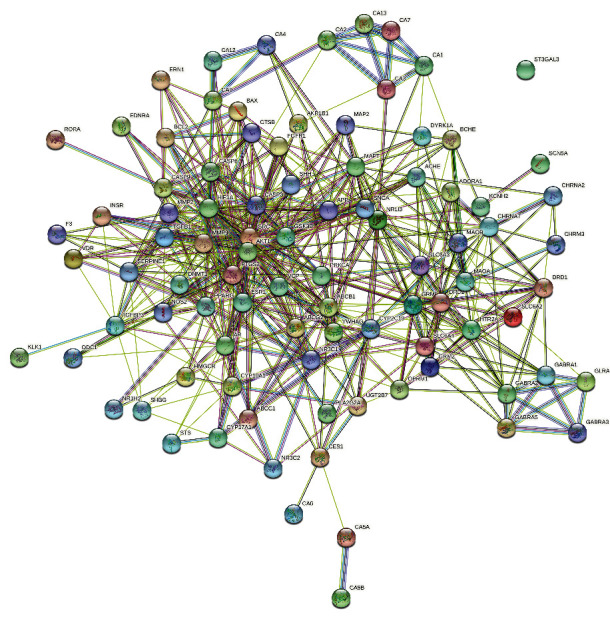
Network diagram of *Abrus cantoniensis*-epilepsy target protein interaction. Each sphere represents a gene target, and the connection between each sphere means the relationship between genes.

**Figure 4:**
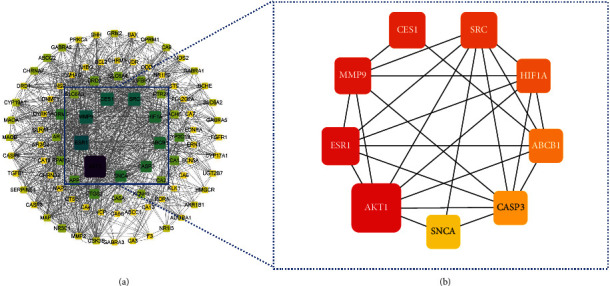
Map of core target. The target near the center of the circle is the core target.

After repeated targets were removed, 174 targets of *Abrus cantoniensis* active ingredients were obtained. Combining the GeneCards database and OMIM database platform, 3170 epileptic targets were obtained. Intersecting the target related to *Abrus cantoniensis* with the disease target, we obtained the Wayne diagram of *Abrus cantoniensis* target for epilepsy treatment (see Figure 2(a)). Finally, 92 common targets were obtained, and the three effective components of *Abrus cantoniensis* were mapped with epilepsy genes (see Figure 2(b)). It was found that there were 5 common targets of three components and epilepsy; 53 action targets of flavanone and epilepsy; and 42 action targets of *β*-sitosterol and epilepsy, among which 29 were corresponding targets of stigmasterol and epilepsy (see Figures 3 and 4). The results indicate that each component of *Abrus cantoniensis* had different effects on epilepsy and could play its role through multiple targets. (see Table 2).

### 3.3. Biological Function and Pathway Analysis

**Figure 5:**
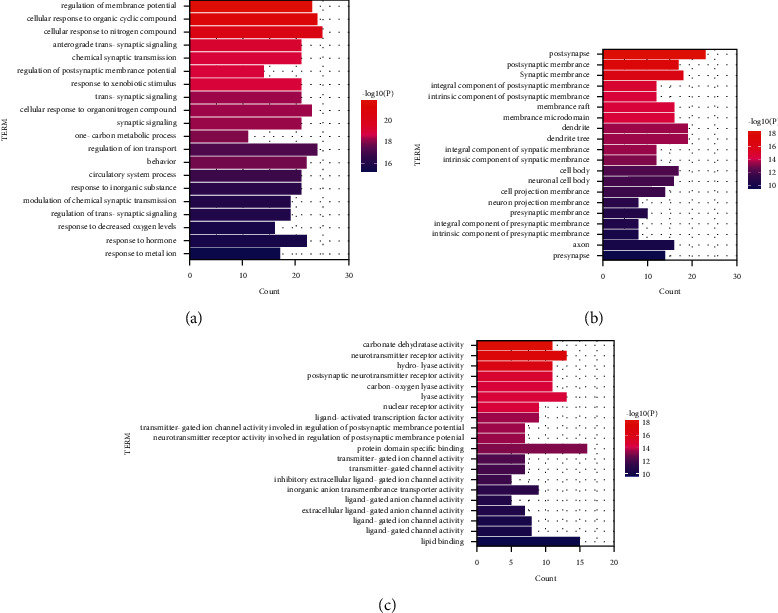
(a) Biological process; (b) cellular component; (c) molecular function.

The results of GO annotation (see Figure 5) showed that the first 20 biological functions of *Abrus cantoniensis*, the key target of epilepsy treatment, were mainly involved in neurotransmitter receptor activity; extracellular ligand-gated ion channel activity molecule function inhibition; neuron projection; presynaptic membrane, postsynaptic membrane and biological membrane potential regulation; organic cyclic compounds reaction; nitrogen compounds reaction; chemical synaptic transmission; the decrease of oxygen level and other biological processes responsiveness. It showed that the effective components of *Abrus cantoniensis* played a role in treating epilepsy by regulating various biological pathways.

**Figure 6:**
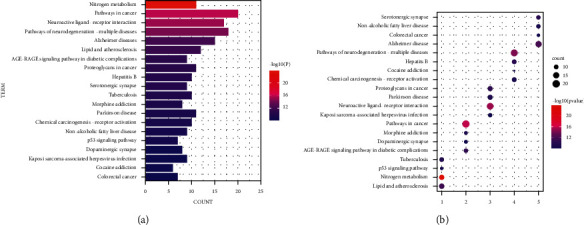
(a) KEGG enrichment network diagram. The gradual change from red to blue represents the increasing *p* value. (b) KEGG bubble diagram. The circle size in the diagram represents the number of enriched genes. The more enriched genes, the bigger the circle. The process of changing from red to blue describes the process of the *p* value from small to large.

The enrichment results of KEGG pathway (see Figure 6) show that the key target genes of *Abrus cantoniensis* in treating epilepsy were significantly enriched in the several signal pathways, e.g., neuroactive ligand-receptor interaction pathway, various neurodegeneration-signal pathway, Alzheimer's pathway, lipid and atherosclerosis pathway, a pathway of proteoglycan in cancer, hepatitis B pathway, serotonergic synapse pathway, *tuberculosis* pathway, morphine addiction pathway, and Parkinson's pathway. The first 20 signaling pathways are also involved, such as the chemical carcinogenesis-receptor activation pathway, nonalcoholic fatty liver pathway, p53 pathway, dopaminergic synapse pathway, Kaposi's sarcoma-associated herpesvirus infection pathway, and cocaine addiction pathway; besides, the first 20 KEGG pathways, GABAergic synapse, HIF-1 pathway, PI3K-Akt pathway, etc., were also included (*p* < 0.05). It is speculated that these pathways may also be in *Abrus cantonensis*.

**Figure 7:**
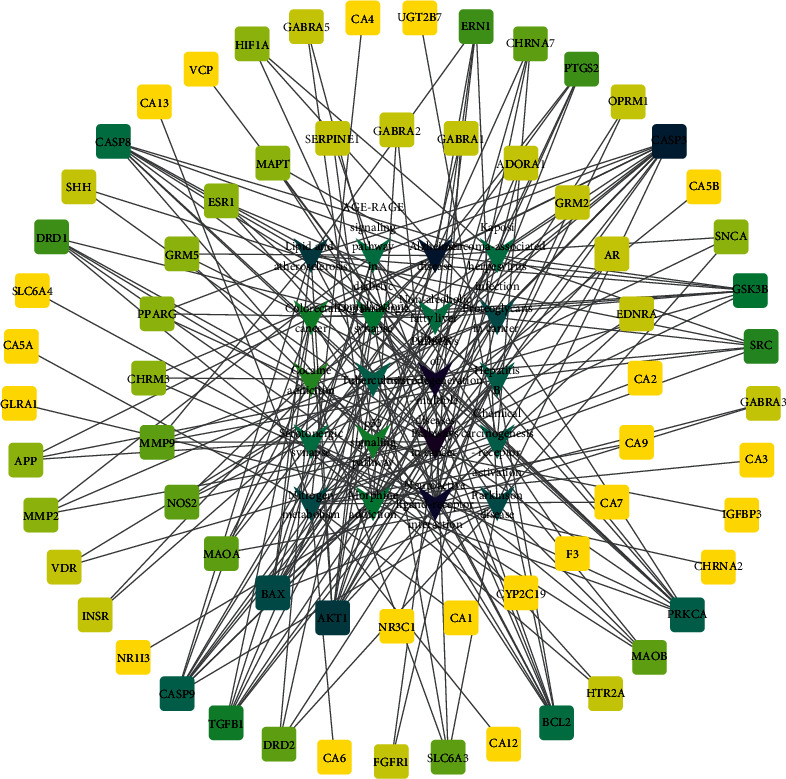
KEGG-target diagram. The inverted V represents the KEGG pathway, and the square represents the target. The darker the square in the diagram, the more genes are involved; the darker the inverted V represents, the more enriched genes.

The first 20 enriched KEGG pathways were calculated using the Cytoscape3.9.0 software, and the degree values of potential targets were predicted. Based on these values, the network diagram of the “target-KEGG pathway” relationship was constructed (see Figure 7). It was found that the main genes in the KEGG network pathway of *Abrus cantoniensis* were highly consistent with the core genes, which demonstrated that *Abrus cantoniensis* might play a role in epilepsy through multiple active components, multiple targets, and multiple pathways.

### 3.4. Construct a Drug-Active Ingredient-Disease-Target Network

**Figure 8:**
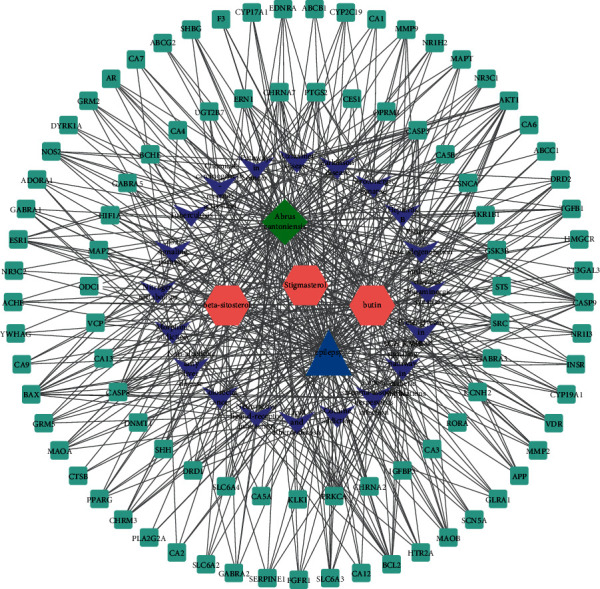
Drug-active ingredient-disease-target network. The green rectangles represent targets, pink hexagons represent pathways, green diamonds represent *Abrus cantonensis*, blue triangles represent epilepsy, and purple inverted vs. represent pathways.

Through relational network analysis, 117 nodes and 525 edges are obtained (see Figure 8). The results showed that *Abrus cantoniensis* mainly acted on 92 targets, which might affect the occurrence and development of epilepsy through three effective components. This manifested that *Abrus cantoniensis* acts on different targets to produce the utility of network regulation and works in treating epilepsy through the interaction of potential targets.

### 3.5. Docking Component-Target Molecule

**Figure 9:**
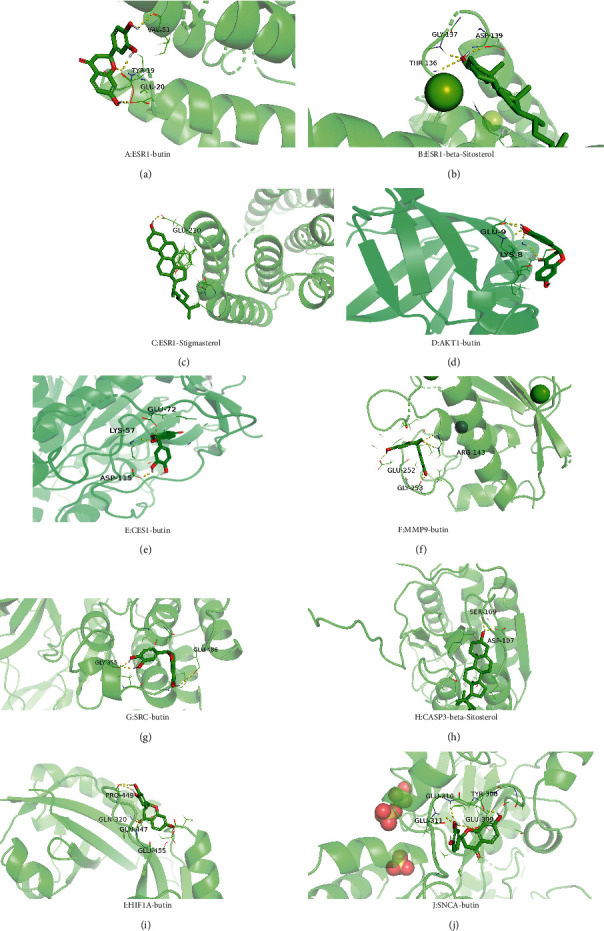
Docking diagram of core target molecules.

According to the nuclear genes obtained by screening, the core genes were molecularly docked with the components (see Table 3). It was found that except ABCB1 gene, all the other core genes were docked with binding energy less than −5 kcal mol^−1^. Core gene ESR1 is docked in *β*-sitosterol, stigmasterol, and flavanone, and the binding energy of gene ESR1 and stigmasterol is −9.13 kcal mol^−1^, which is the smallest among all core genes, indicating that the binding activity of the two genes is good. Among three components, flavanone is involved in the seven cores' docking process. Hence, we speculated that *Abrus cantoniensis* is closely related to epilepsy through flavanone. See Figure 9 for a specific docking form.

## 4. Discussion

Through gene database searches and the “drug-active ingredient-disease-target” network constructs, we concluded three main active ingredients, including *β*-sitosterol in *Abrus cantoniensis*; screened out 174 targets with the high possibility related to the ingredients; obtained 3170 potential targets for epilepsy; found 92 targets at the intersection of *Abrus cantoniensis* and epilepsy. Moreover, the above network diagram intuitively showed that the drug has multiple components and targets.

*β*-sitosterol has an obvious anticonvulsant effect among the main active ingredients of *Abrus cantoniensis* [9, 10]. Also, *β* -sitosterol exerts a neuroprotective effect on hippocampal neurons with epileptic discharge [7]. Stigmasterol-treated animals show a significant decrease in exercise activity, rigid behavior, immobility duration, and an increase in latency. Later biochemical evaluation showed that GABA and GSH levels increased, while dopamine, MDA, TNF-*α* of levels, and AChE activity decreased. Histopathological changes in the cerebral cortex confirmed these findings [11]. It is speculated that stigmasterol may help reduce seizures. The above results are similar to our study, suggesting that the effective components of *Abrus cantoniensis* have some antiepileptic effects. *Abrus cantoniensis* mainly affects epilepsy occurrence through multiple targets and multiple signal pathways. There might be a synergistic effect between these targets and pathways. The core targets of *Abrus cantoniensis* in epilepsy treatment includes AKT1, ESR1, MMP9, CES1, SRC, HIF1A, ABCB1, CASP3, and SNCA.

Wang et al. [12] observed epileptic rats and found the effect of catgut embedding at acupoint on cognition and the PI3K-AKT signal pathway: activating the PI3K-AKT signaling pathway helped reduce hippocampal neurons' damage and improve cognition. Zheng et al. [13] found that activating AKT inhibits the activity of GSK3*β* and inactivates it. AKT also plays an important role in protecting neurons and improving learning and memory because it can resist cerebral ischemia, hypoxia, and hippocampal neuron injury, preventing neuron apoptosis. Seizures during epilepsy significantly inhibit the plasticity of synapses, and the short-term plasticity mainly depends on the fluctuation and steady state of calcium levels in synapses [14]. The neuroprotective effect of ESR1 mainly manifests in its influence on synaptic plasticity, which explains the role of ESR1 in epilepsy treatment [15]. Xiao et al. [16] suggested activation of the ASK1-MMP9 signal is critical in the pathological process of temporal lobe epilepsy. They found that the phosphorylation of ASK1 and the expression of MMP9 in hippocampus significantly increased in temporal lobe epilepsy, and the upregulation of MMP9 expression was caused by activation of ASK1. Zheng et al. [17] found that the expression of MMP9 in a rat's brain affected the immune response, caused changes in neuronal reactivity and synaptic remodeling in the hippocampus, regulated the excitability of nerve cells, and participated in the immunological pathogenesis of epilepsy. Sanna et al. [18] observed and proved the role of PTKs in the SRC family in epilepsy pathophysiology, which might be the potential therapeutic target of antiepileptic therapy. ABCB1 protein affects the drug resistance and blood drug concentration of epilepsy patients. Yue [19] found that different genotypes of ABCB1 rs1002204 and rs10234411 loci related to the therapeutic effect of AEDs. Ru et al. [20] found after epileptic patients took carbamazepine orally, the gene polymorphism of the SNP site G2677T/A in ABCB1 gene impacts the blood drug concentration suggesting that patients with TT genotype can reduce the drug dosage appropriately. In epilepsy, with neuronal apoptosis, the expression of caspase-3 increases. Ding et al. [21] further verified that caspase-3 participates in neuronal apoptosis caused by epilepsy. Serotonin 5-hydroxytryptamine is effective regulation of neuronal excitability. Long-term studies have shown that it can control the seizure activity of epilepsy. Animal models show that the basic level and turnover rate of 5-hydroxytryptamine in the hippocampus of epileptic rats is lower than that of normal rats, and the transmission of 5-hydroxytryptamine is impaired [22]. Using glutamate or bicuculline (GABA-A receptor antagonist) in a rat's dorsal medial thalamic nucleus prolongs the electrophysiological seizure, while using GABA-A receptor agonist shortens the seizure time [23]. To sum up, the genes of *Abrus cantoniensis* are closely related to epilepsy.

KEGG signal pathway and GO biological process enrichment analysis on *Abrus cantoniensis* therapeutic target was carried out to further analyze the signal pathway and biological process involved in *Abrus cantoniensis* therapeutic target. It was found that the genes of *Abrus cantoniensis* for treating epilepsy were engaged in many KEGG pathways. Kong et al. [24] found that by inhibiting the desensitization of AMPA receptors and nerve transmission of GABA, CTZ caused neuron overexcitation and led to seizures, which suggests the GABA signaling pathway interferes with the occurrence of epilepsy. The hepatitis B pathway regulates transcription, cell signal cascade, proliferation, differentiation, and apoptosis. It has been reported that nicotine addiction pathways can participate in nerve transduction, immunity, and metabolism and have a wide range of influences on both the central nervous and peripheral nervous system [25]. In addition, the signal pathway of hypoxia-inducible factor 1(HIF-1) closely relates to neural stem cells' function [26]: HIF-1 adapts to different oxygen levels by regulating its targets' expression; the precise control of oxygen concentration provides a breakthrough for the nervous system diseases' treatment [27]. Wang et al. [28] found that the p53 gene plays a vital role in neuronal apoptosis after pentylenetrazol-induced status epilepticus in rats. In addition, Xia et al. [29] found that the p38 inhibitor has a particularly protective effect on neuronal damage in SE rats, and its mechanism is related to blocking the p53-mediated apoptosis pathway.

Biological processes in our study are all related to the occurrence and metastasis of epilepsy; both oxidative stress and neuronal apoptosis are related to seizures. In animal epilepsy models, antioxidation therapy reduces the nerve damage caused by oxidative free radicals, thus playing an antiepileptic role [30, 31]. The synapse's function is closely related to epilepsy [32]. Most antiepileptic drugs could regulate voltage-gated ion channels, enhance the inhibition mediated by C-aminobutyric acid (GABA), regulate synaptic release, or block ionic glutamate receptors. Studies [33] show that the current AEDs mainly regulate neurotransmitters' balance by acting on ion channels, transporters, and receptors to relieve symptoms. For example, the antagonist of *α*-amino -3-hydroxy-5-methyl-4- isoxazole propionic acid receptor (AMPAR), one of glutamate receptors, can significantly reduce or eliminate the epileptiform activity of in vitro preparations. In addition, injection of dopaminergic D1/D2 drugs into the back of the striatum has been proven to inhibit seizures. In contrast, injection of GABA-A agonist and N-methyl-d- aspartate (NMDA) antagonist into substantia nigra also inhibits absence seizures. Inflammation and oxidative stress play an important role in the occurrence and drug resistance of epilepsy, e.g., immunosuppressive corticosteroids and ketogenic diet play an antiepilepsy by suppressing inflammatory signals, reducing reactive oxygen species, or protecting/restoring the blood-brain barrier [34]. At the same time, the signal pathway of cancer obtained by KEGG enrichment analysis in this study is related to the onset of epilepsy. It is suggested that *Abrus cantoniensis* treats epilepsy through multiple signaling pathways, mainly involving various complex biological processes such as cell differentiation and apoptosis, metabolism, inflammation, and immunity. It embodies the characteristics of *Abrus cantoniensis* with multiple components, multiple targets, and multiple ways to treat epilepsy. All in all, the enrichment analysis of *Abrus cantoniensis* therapeutic target, KEGG signal pathway, and GO biological process in our study are consistent with the previous results on the effective active ingredients in *Abrus cantoniensis*.

## 5. Conclusion

To sum up, this study reveals the potential mechanism of *Abrus cantoniensis* in the treatment of epilepsy through network pharmacology. We found that *Abrus cantoniensis* may inhibit epilepsy occurrence by reducing neuronal damage, increasing blood drug concentration, reducing drug resistance, shortening seizure time, prolonging seizure cycle, etc., which can serve as a reference in epilepsy treatment. However, network pharmacology analysis only provides a theoretical prediction, which needs to be verified by further experiments and more clinical observations.

## Figures and Tables

**Table 1:** Active ingredients of *Abrus cantoniensis*.

Mol ID	Ingredients	OB/(%)	DL	Structure
MOL000358	Beta-sitosterol	36.91	0.75	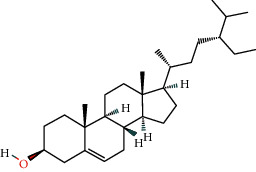
MOL000449	Stigmasterol	43.83	0.76	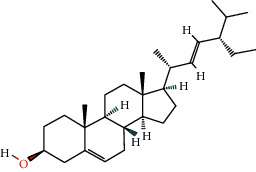
MOL002975	Butin	69.94	0.21	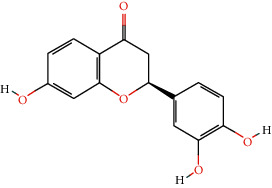

**Table 2:** Information of core targets.

Number	Protein	Gene	UniProt ID	Ingredient
1	Serine/threonine-protein kinase AKT	AKT1	P31749	Butin
2	Estrogen receptor alpha	ESR1	P03372	Butin, stigmasterol, beta-sitosterol
3	Matrix metalloproteinase 9	MMP9	P14780	Butin
4	Acyl coenzyme A: cholesterol acyltransferase	CES1	P23141	Butin
5	Tyrosine-protein kinase SRC	SRC	P12931	Butin
6	Hypoxia-inducible factor 1 alpha	HIF1A	Q16665	Butin
7	P-glycoprotein 1	ABCB1	P08183	Butin
8	Caspase-3	CASP3	P42574	Beta-sitosterol
9	Alpha-synuclein	SNCA	P37840	Butin

**Table 3:** Docking information of core target molecules, in which the unit of binding energy is kcal mol^−1^.

Number	Gene name	Ingredient	Docking position	Binding energy
1	AKT1	Butin	GLU-9, LYS-8	−6.15
2	ESR1	Butin	VAL-51, TYR-19, GLU-20	−7.22
3	ESR1	Stigmasterol	GLU-210	−9.13
4	ESR1	Beta-sitosterol	GLY-137, ASP-139, THR-136	−6.08
5	MMP9	Butin	GLU-252, ARG-143, GLY-253	−8.47
6	CES1	Butin	GLU-72, LYS-57, ASP-115	−6.99
7	SRC	Butin	GLY-355, GLU-486	−7.05
8	HIF1A	Butin	PRO-449, GLN-320, GLN-447, GLU-455	−6.13
9	CASP3	Beta-sitosterol	SER-109, ASP-107	−7.17
10	SNCA	Butin	GLU-309, GLU-310, GLU-311, TYR-308	−7.65

## Data Availability

The datasets analyzed during the current study are available from the corresponding author upon reasonable request.
